# Nurses' Wages Fixed by a Dutch Auction

**Published:** 1887-02-12

**Authors:** 


### WORKHOUSE NURSING: HOW NOT TO DO IT.

### NURSES' WAGES FIXED BY A DUTCH AUCTION.

WE venture to call the attention of Mr. Ritchie, the President of the Local Government Board, to the proceedings of the Guardians of the Helston Union as reported in *The Cornish Telegraph* of the 27th ult., to which we are indebted for the following account. Will not the Local Government Board cause an official inquiry to be made into the condition of the sick paupers in the Helston workhouse? as it is necessary that this step should be taken to protect the suffering from a vicious system. The Workhouse Infirmary Nursing Association will, we hope, take up the question also, and create a wholesome public opinion on the subject in Helston and the neighbourhood. Fancy asking a nurse (?)—save the name—to bargain with a Board of Guardians as to the rate of her remuneration, and then paying her £13 per annum, or only half the wages an efficient nurse receives all over the country.

"Another long, acrimonious, and personal discussion arose at the meeting of the Helston Guardians on Saturday, over the question of the appointment of a nurse to the Workhouse Infirmary. Sarah Vincent, a widow of forty, living near to the workhouse at Helston, and whose character was unimpeachable, offered her services for £15 a year; Mrs. Gill, of Lemon Row, Truro, late a nurse at St. Thomas's Workhouse, Exeter, was another applicant, but asked £18 a year; and Mrs. Stephens, who was known personally to the Guardians as a most respectable person, asked £18 per annum. Fanny Moyle's name was not before the Board, other than by her previous application, and only Mrs. Vincent and Mrs. Stephens were in attendance to answer any questions which might be put by the Guardians. Mrs. Vincent said that she would be willing to enter upon the duties at a salary of £13, but Mrs. Stephens would not budge from the amount she had named in the first instance. The Rev. W. H. Bloxsome proposed, and Mr. Glasson seconded, the appointment of Mrs. Vincent; the rev. gentleman, in reply to a remark from Mr. W. H. Thomas, declaring that Fanny Moyle was disqualified. This remark Mr. W. H. Thomas strongly resented. Canon Tyacke asked that Mr. Dunstan should be requested by the chairman to give his candid opinion, as the only man who had known Fanny Moyle for a long number of years, as to that person's character and efficiency. The request was not pressed, as several Guardians objected to the form in which it was put, and declared that Fanny Moyle was a 'reformed character.' Mr. Ellis said that if Fanny Moyle had been present, he should have asked her a few questions, but, as she was not, he should refrain from saying anything whatever. This remark of Mr. Ellis called forth angry retorts from Mr. Pearce and other members of the Board, but Mr. Carter Thomas ventured the assertion that Moyle's past history disqualified her from accepting any office in the House. In the office of nurse, they should have a woman whom the inmates of the Infirmary could respect. Several pointed personalities, reflecting on the position Canon Tyacke had taken up, ensued, and Mr. Ellis said that the ratepayers would not care to consent to the appointment of Fanny Moyle. If the Guardians appointed her, they would afterwards find that the minority who voted against the proposal was in the right. Mr. W. H. Thomas proposed, and Mr. W. Rowe seconded, Fanny Moyle's appointment at £13 a year, and this resolution was carried by nineteen votes to thirteen. This number constituted more than half the Guardians, and the Clerk therefore ruled that the woman had been duly elected. Canon Tyacke entered a protest against this proceeding, and informed the Board that he should communicate with the Local Government Authority. Mr. Pearce hoped that the Guardians would take up a strong stand against the Local Government Board, should that body not approve of the appointment."

